# Evaluation of the guaiac fecal occult blood test for detection of gastrointestinal bleeding in the rhesus macaque (*Macaca mulatta*)

**DOI:** 10.1111/jmp.12446

**Published:** 2019-11-01

**Authors:** Rachel Elizabeth Cooper, Eric Kenneth Hutchinson, Jessica Marie Izzi

**Affiliations:** ^1^ Department of Molecular and Comparative Pathobiology Johns Hopkins University School of Medicine Baltimore MD USA

**Keywords:** colon, duodenum, fecal occult blood testing, guaiac fecal occult blood testing, hemorrhage, non‐human primate, stomach, tissue biopsy

## Abstract

**Background:**

Gastrointestinal (GI) hemorrhage accompanies several common diseases of rhesus macaques (*Macaca mulatta*). Guaiac fecal occult blood testing (gFOBT) is a non‐invasive means to detect such bleeding in several species; however, there are currently no data indicating reliability of this test to detect GI hemorrhage in macaques.

**Methods:**

We evaluated sensitivity and specificity of gFOBT to detect simulated and biopsy‐associated bleeding in the stomach, duodenum, and colon of 15 rhesus macaques. Fecal samples were analyzed via gFOBT for 72 hours.

**Results:**

Guaiac fecal occult blood testing was more sensitive to detect lower vs upper GI bleeding; sensitivity was volume‐dependent in the upper GI tract. Single‐test specificity was 95.2%. Repeated fecal collections increased gFOBT sensitivity without affecting specificity.

**Conclusions:**

Guaiac fecal occult blood testing is a useful screening test for both upper and lower GI bleeding in rhesus macaques. For highest sensitivity, gFOBT should be performed on three fecal samples collected 24 hours apart.

## 1 INTRODUCTION

A current challenge in non‐human primate medicine is early and accurate diagnosis of gastrointestinal (GI) tract diseases, many of which are associated with GI tract bleeding. For example, neoplasms of the GI tract in macaques have been reported in conjunction with gross findings of hemorrhagic ulceration,^1^, ^2^ clinical signs of hematochezia or melena,^3^ and microcytic hypochromic anemia.^4^ All of these findings indicate that bleeding in the GI tract is an important sign of GI neoplasia. In rhesus macaques (*Macaca mulatta*), the most common GI tract neoplasm—adenocarcinoma—has a 20%‐30% incidence in individuals over 30 years of age and a metastatic rate of 34% at the time of diagnosis.^3^ GI adenocarcinoma is most commonly located in the ileocolic junction, cecum, colon, and jejunum, though it has also been identified in the duodenum and ileum.^1^, ^3^, ^5^, ^6^ Early diagnosis of intestinal adenocarcinoma is associated with improved surgical outcomes and long‐term prognosis,^1^, ^3^ making accurate methods of detection of utmost importance.

Non‐neoplastic disease processes that may cause GI bleeding are also prevalent in macaques and have the potential to compromise animal welfare and research validity. Shigellosis and campylobacteriosis, both common in rhesus macaques, can present with intestinal bleeding.^7^, ^8^ Additionally, diverticulosis and idiopathic enterocolitis, shown to cause GI bleeding in humans,^9^ have been reported in rhesus macaques.^10^, ^11^ Chronic idiopathic diarrhea, secondary to enterocolitis in rhesus macaques, has been reported with incidence of up to 15% in breeding colonies of macaques.^12^ Also in rhesus macaques, gastric ulcers have been reported in association with chronic stress,^13^ as well as in apparently spontaneous cases.^14^ Experimental gastric infection with *Helicobacter pylori*, which is enzootic in many macaque populations, has also been shown to cause gastritis,^15^, ^16^ as has infection with *Shigella flexneri*.^17^ While not specifically reported in macaques, gastritis is associated with gastric bleeding in humans.^18^ In all of the aforementioned diseases, identification of bleeding within the GI tract may be critical for diagnostic, prognostic, and treatment purposes.

Fecal occult blood testing (FOBT) is used commonly in human medicine as an early, non‐invasive, diagnostic aid in cases of anemia or signs of GI disease; it is also used to screen for colorectal cancer.^19^ Positive test results suggest the presence of GI hemorrhage and prompt further diagnostic procedures to localize the bleed and arrive at a diagnosis.^20^ There are currently three primary FOBT classifications: chromogen‐based, immunochemical (iFOBT), and fluorometric (fFOBT). Guaiac‐type tests (gFOBT), which are chromogen tests, are commonly used in veterinary as well as human medicine^19^, ^21^, ^22^ and depend on the presence of the intact heme moiety to catalyze oxidation of guaiac when hydrogen peroxide is added. An informal survey of institutions housing primates revealed that the gFOBT is the most common FOBT used by non‐human primate clinicians. Though several case reports reference use of FOBT—presumably gFOBT—in diagnosing GI disease in macaques,^3^, ^4^, ^16^ the validity of gFOBT has not previously been rigorously evaluated in any macaque or other NHP species. In addition, we are not aware of an iFOBT test specific for any non‐human hemoglobin sequence, and fFOBT assays are not widely available in laboratories that accept non‐human samples. Therefore, we focused this study on evaluation of gFOBT as a diagnostic tool for use in rhesus macaques.

Given the prevalence and impact of GI adenocarcinoma and other GI diseases in rhesus macaques, as well as the lack of data describing usefulness of gFOBT in cases of suspected GI bleeding, we sought to develop guidelines for gFOBT use and interpretation in this species. In humans, gFOBT has been shown to be less sensitive to detect upper GI bleeding as compared to lower GI bleeding and less sensitive to detect smaller as compared to larger blood volumes.^23^, ^24^, ^25^ The current recommendation in humans is for gFOBT to be performed using samples from three consecutive bowel movements, as repeated testing has been shown to increase sensitivity. Based on these data, we hypothesized that in rhesus macaques (a) gFOBT detects bleeding in the upper and lower GI tract, (b) sensitivity of gFOBT is higher for lower as compared to upper GI bleeding, and (c) sensitivity of gFOBT is higher at larger blood volumes. In order to evaluate these hypotheses, we induced simulated and biopsy‐associated hemorrhage in the stomach, duodenum, and colon of rhesus macaques. We then used a commercially available gFOBT (OneStep™ + ER, FOB Enhanced Readability Test Kit, Henry Schein^®^) to detect blood in the feces at different time points. Additionally, in order to develop recommendations for testing frequency, we evaluated the effect of repeated testing regimens on sensitivity of gFOBT in rhesus macaques.

## 2 MATERIALS AND METHODS

### 2.1 Humane care guidelines

This protocol was approved by the IACUC of Johns Hopkins University, an AAALAC‐accredited institution. All procedures were conducted in accordance with the US National Research Council's *Guide for the Care and Use of Laboratory Animals*,^26^ the US Public Health Service's Policy on Humane Care and Use of Laboratory Animals,^27^ and the US Department of Agriculture's Animal Welfare Regulations.^28^

### 2.2 Animals

Subjects were 15 Chinese‐origin male rhesus macaques (*Macaca mulatta*; age 14; weight 9.0‐16.4 kg); this cohort has previously been described.^29^ Macaques selected for use in this study were singly housed for reasons unrelated to this study. All macaques were fed a standard commercial diet (2050 Teklad Global 20% Protein Primate Diet, Harlan Laboratories). Commercial diets were supplemented 5 days per week with a variety of food enrichment items, including foraging mix, nuts, and produce (fresh and dried). Water was freely available via an automatic watering system.

### 2.3 Study design

All macaques enrolled in the study were considered to be in good clinical health. Inclusion criteria were an unremarkable physical exam, complete blood count, and serum chemistry (assessed within 2 months prior to study initiation, and subsequently semiannually); seronegativity for simian immunodeficiency virus, simian retroviruses 1 and 2, simian T‐lymphotropic virus, and macacine herpesvirus 1; and a negative tuberculin skin test for *M tuberculosis* within the previous 6 months. Macaques were either vaccinated against (n = 10) or seronegative for (n = 5) measles virus. Exclusion criteria were history of chronic disease; history of anemia (hematocrit < 32%)^30^; signs of gastrointestinal disease within the past 12 months (eg, diarrhea, weight loss, hematochezia, melena, constipation); NSAID or anticoagulant drug use within the past 12 months; and female sex, to avoid false‐positive gFOBT results associated with menstruation. Peroxidase‐rich produce (eg, horseradish, cantaloupe, turnip, broccoli, cauliflower, red radish, parsnip) was avoided for the duration of the study, as this has been shown to produce false‐positive results.^31^ All macaques were fasted overnight prior to each study procedure.

**Figure 1:**
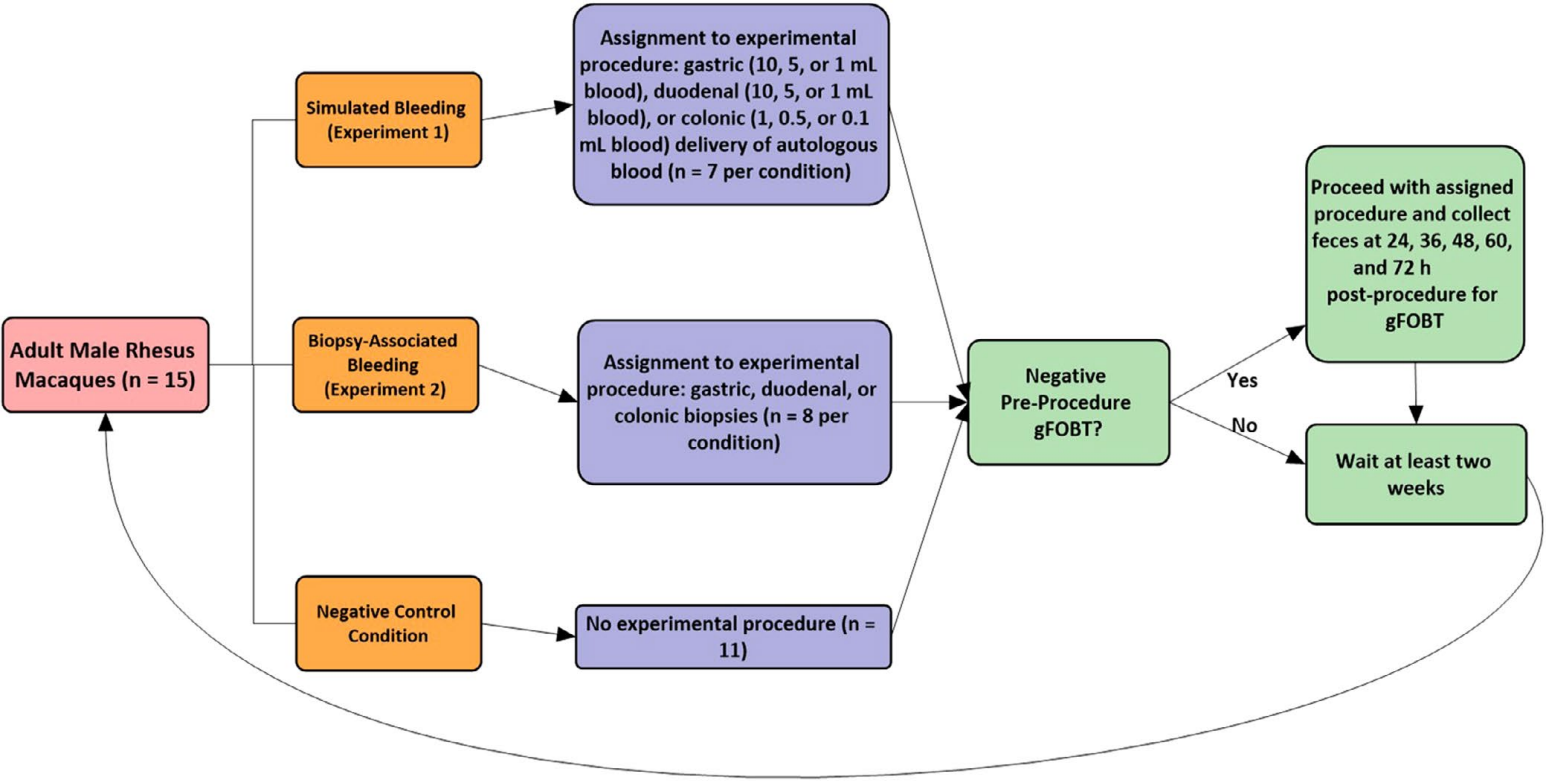
Experimental timeline

Two experiments were performed (Figure 1). In both experiments, macaques were assigned to multiple experimental conditions with a washout period of at least 13 days between each condition. Experiment 1 was conducted first, with Experiment 2 and the control condition occurring after conclusion of Experiment 1. Within each experiment, experimental conditions were applied in an interspersed manner. On each day that study procedures were to be performed, exclusion criteria were applied and remaining available animals were ad hoc randomly assigned to one of the experimental conditions (and corresponding procedure) available to be performed that day. In Experiment 1, no macaque underwent any experimental condition more than once. In Experiment 2, four macaques each underwent a single experimental condition two times. In addition to these two experiments, 11 macaques were assigned to a control group used to establish specificity of gFOBT (see Fecal Collection).

Two pre‐procedural fecal samples were collected on the morning of each experimental procedure; animals with a positive pre‐procedural gFOBT were excluded from the study on that day. Throughout the study, seven such positive results were obtained during pre‐testing. These positive pre‐test results were obtained 13 to 27 days following prior procedures, representing 7.9% of Experiment 1 pre‐tests and 8.3% of Experiment 2 pre‐tests.

### 2.4 Experiment 1: Simulated hemorrhage (autologous blood administration)

Animals were assigned to multiple of nine groups (n = 7 per group): 1, 5, or 10 mL autologous blood delivered to the stomach (groups 1‐3) or duodenum (groups 4‐6); and 0.1, 0.5, or 1.0 mL autologous blood delivered to the colon (groups 7‐9). Experimental blood volumes were selected on the basis of estimated physiological and clinical relevance in relation to data from human subjects.^23^ No volumes of blood greater than 1 mL were instilled into the colon because of the high likelihood of presenting as frank blood in feces, negating the need for a fecal occult blood test. At the start of each procedure, 0.1‐10 mL of blood, according to experimental group, were collected directly into a heparinized syringe (10 U heparin sodium/mL blood) from the femoral vein of each macaque under chemical restraint with ketamine (10‐15 mg/kg IM; Zetamine; VetOne). Procedures for each group subsequently differed as follows:

#### 2.4.1 Simulated gastric hemorrhage

Immediately after the blood collection, autologous heparinized blood was administered directly into the stomach via an orogastric tube, which was flushed using a volume of sterile saline approximately 1 mL greater than the tube's capacity prior to tube withdrawal and recovery of the macaque.

#### 2.4.2 Simulated duodenal hemorrhage

An intravenous catheter was placed in the cephalic vein. Tracheal intubation was facilitated by intravenous administration of propofol (up to 4 mg/kg; PropoFlo 28; Zoetis, Parsippany‐Troy Hills, NJ), and anesthesia was maintained with isoflurane (0%‐2.5%; Forane, Baxter Healthcare) delivered via a precision vaporizer throughout the remainder of the procedure. A flexible fiber gastroscope (diameter, 7.9 mm; channel, 2.0 mm; field of view, 100°; GIF XP20, Olympus) was advanced via the oral cavity into the proximal duodenum, with insufflation of the stomach as needed. Blood was administered to the duodenum via the endoscope and flushed using a volume of sterile saline approximately 1 mL greater than the capacity of the endoscope channel. To minimize inadvertent removal of delivered blood, the endoscope was left in place for 5 minutes and directed away from the blood delivery site prior to desufflation, endoscope withdrawal, and conclusion of anesthesia.

#### 2.4.3 Simulated colonic hemorrhage

Blood was immediately administered directly into the colon via an eight French red rubber catheter digitally advanced to a depth of 15‐20 cm, which corresponds to the pelvic colon in the adult rhesus macaque.^32^ Following blood administration, the catheter was flushed using a volume of sterile saline 1 mL greater than its capacity prior to catheter withdrawal and recovery of the macaque.

### 2.5 Experiment 2: Biopsy‐induced hemorrhage

Animals were assigned to three groups (n = 8 per group): (a) gastric biopsy, (b) duodenal biopsy, and (c) colonic biopsy. For animals in the gastric or duodenal biopsy group, anesthesia was induced and maintained as described for the simulated duodenal hemorrhage group (Experiment 1). A flexible endoscope was advanced via the oral cavity into the stomach or the proximal duodenum, and biopsy forceps were inserted into the stomach or duodenum via the endoscope. Three partial‐thickness pinch biopsies were taken from unique sites in the body of the stomach or the proximal duodenum (at least 5 cm past the pyloric sphincter) according to experimental group, and sites were observed to confirm bleeding prior to withdrawal of the endoscope and conclusion of anesthesia. For the colonic biopsy group, three partial‐thickness pinch biopsies were obtained blindly from each animal by inserting biopsy forceps to an approximate depth of 15‐20 cm from the anorectal junction.^11^, ^33^

### 2.6 Fecal collection

Two fecal samples per animal were collected from the cage pan at the following time points: 24, 36, 48, 60, and 72 hours post‐procedure. Following sample collection, the cage pan was cleaned and disinfected with Quatricide PV‐15 (Pharmacal Research Laboratories, Inc) and then rinsed (following the 24‐ and 48‐hour collections) or cleared of gross fecal material (following the 36‐ and 60‐hour collections). For the 11 control animals, fecal samples were collected on the same timeline as for experimental conditions, but no experimental procedure was performed. If multiple fecal boluses were present, sampling comprised fecal aliquots from two boluses. If only one bolus was present, sampling comprised an aliquot from each end of the bolus. If no fecal sample was present, no sample was collected at that time point.

Across all conditions, comprising 98 total experimental trials, only 14 trials resulted in fecal collection and gFOBT testing at all five post‐procedural time points. All trials resulted in at least two collections. There was no difference in fecal production between sites of simulated or induced GI bleeding (including controls), as assessed by number of post‐procedural time points in which feces were available for gFOBT (Kruskal‐Wallis test, *P* = .99).

### 2.7 gFOBT Procedure

**Figure 2:**
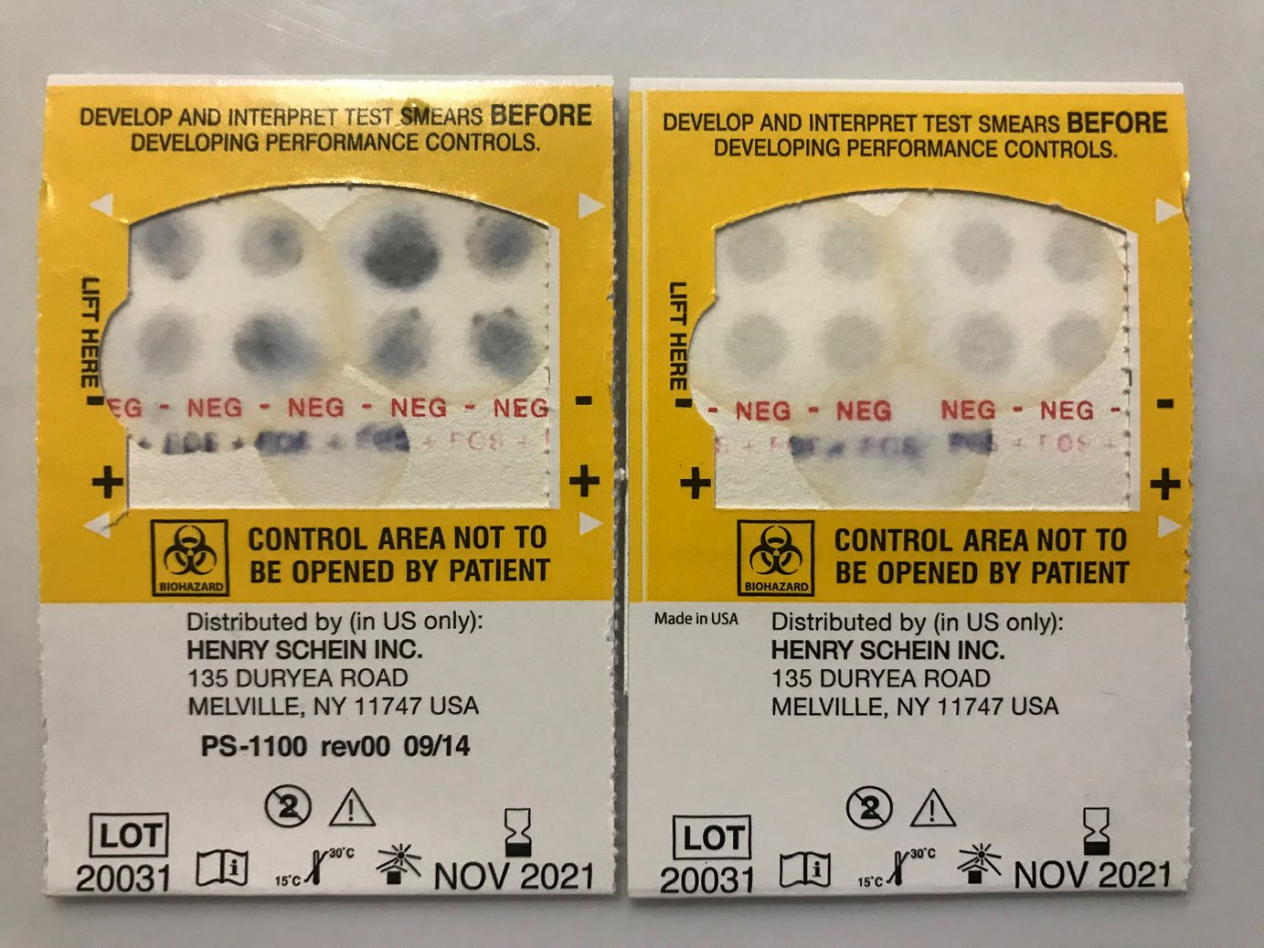
Example of a positive (left) and negative (right) guaiac fecal occult blood test (gFOBT), following development of the test paper with hydrogen peroxide

Guaiac fecal occult blood testing was performed at room temperature following sample collection. One fecal aliquot from each of the two samples collected per animal was smeared onto a test card using a wooden applicator, according to the manufacturer's instructions (OneStep™ + ER, FOB Enhanced Readability Test Kit, Henry Schein^®^). Results were interpreted as positive or negative 45‐60 seconds after addition of peroxide; gFOBT was interpreted as positive for any given time point if at least one of the two fecal aliquots was positive (Figure 2). A single individual conducted and interpreted all gFOBT tests. The interpreter was blinded to treatment group for as many samples as achievable (85% of all gFOBT tests); blinding was not possible on days in which fecal samples from only one experimental group were available. In comparing blinded vs unblinded gFOBT tests for experimental groups in which unblinded tests had been performed, there was no difference in correspondence of gFOBT results with expected results (ie, positive gFOBT result following an experimental condition, and negative gFOBT result following a control condition) (Odds ratio 0.72, 95% CI 0.39‐1.33).

### 2.8 Statistical analysis

All statistical analyses were performed using VassarStats (vassarstats.net) or GraphPad Prism version 7.00 for Windows (GraphPad Software). For all applicable tests, a *P* value of <.05 was considered significant.

Sensitivity of gFOBT was calculated as the percentage of post‐procedure macaques with at least one positive gFOBT test in the sampling period. Specificity of gFOBT was calculated as the percentage of control group macaques with only negative gFOBT tests in the sampling period. Fisher's exact tests or Fisher‐Freeman‐Halton exact tests were used to analyze the effect of two independent variables—GI segment and volume of blood delivered (or biopsy)—on the binary dependent variable of gFOBT test outcome.

Kruskal‐Wallis tests were used to evaluate whether there was a difference in fecal output or gFOBT sensitivity over multiple time points between different experimental groups. As applicable, a post hoc Dunn's multiple comparison test was used.

To produce practical guidelines for execution and interpretation of gFOBT in such a way as to maximize sensitivity under conditions mimicking clinical disease, data from the biopsy‐induced hemorrhage experiment were pooled for analysis. gFOBT sensitivity and specificity were calculated according to several fecal collection protocols: any single fecal collection (1 sample), any two samples collected 12 hours apart (2 × 12), any two samples collected 24 hours apart (2 × 24), and any three samples collected 24 hours apart (3 × 24). Any one positive sample in a given protocol led to interpretation of the test series as positive. For an animal that provided the maximum possible five post‐procedure fecal samples, analysis included five “1 sample” data points, four “2 × 12” data points, three “2 × 24” data points, and one “3 × 24” data point.

## 3 RESULTS

### 3.1 gFOBT sensitivity and specificity

The gFOBT detected blood originating from all GI segments. Following autologous blood administration or biopsy, the sensitivity of gFOBT to detect blood in at least one of up to five tests was 64% across GI segments and blood volumes (or biopsy), significantly greater than the false‐positive rate of 18% (Fisher's exact test, *P* = .00; Table 1). Specificity of this test was 82% when up to five tests were performed at 12‐hour intervals (Table 1).

**Table 1:** Guaiac fecal occult blood test (gFOBT) specificity following autologous blood administration (n = 7 per condition) to or pinch biopsies (n = 8 per condition) of the stomach, duodenum, or colon in adult male rhesus macaques

Experimental group	Stomach				Duodenum				Colon				Negative Control
	10 mL	5 mL	1 mL	Biopsy	10 mL	5 mL	1 mL	Biopsy	1 mL	0.5 mL	0.1 mL	Biopsy	
gFOBT sensitivity (% positive)	7/7 (100%)	6/7 (86%)	2/7 (29%)	2/8 (25%)	7/7 (100%)	6/7 (86%)	2/7 (29%)	3/8 (38%)	4/7 (57%)	4/7 (57%)	6/7 (86%)	7/8 (88%)	
95% CI	65%‐100%	49%‐99%	5%‐64%	4%‐59%	65%‐100%	49%‐99%	5%‐64%	14%‐69%	25%‐84%	25%‐84%	49%‐99%	53%‐99%	
gFOBT specificity (% negative)													9/11 (82%)
95% CI													52%‐97%

Note

When present in the cage pan, feces were collected for gFOBT every 12 h beginning 24 h and ending 72 h post‐procedure. If at least one positive gFOBT result was obtained, macaques were considered gFOBT‐positive. gFOBT specificity was determined by evaluating negative control animals, in which no procedure was performed prior to fecal collection. Where relevant, sensitivity or specificity is reported with a 95% confidence interval (95% CI).

### 3.2 gFOBT detection of simulated GI bleeding

Across volumes and GI segments, 70% of animals with simulated bleeding tested positive via gFOBT. There were three fecal samples collected for gFOBT that contained grossly visible blood; each of these instances occurred following blood administration to the colon, and in each case, the sample tested positive by gFOBT**.**

#### 3.2.1 Effect of blood volume on gFOBT detection of simulated bleeding in the stomach

One milliliter of blood delivered to the stomach was detected by gFOBT with 29% sensitivity, 5 mL was detected with 86% sensitivity, and 10 mL was detected with 100% sensitivity (Table 1). Sensitivity of the gFOBT to detect blood varied significantly with delivered blood volume (Fisher‐Freeman‐Halton exact test, *P* = .02), with increased detection of larger blood volumes.

#### 3.2.2 Effect of blood volume on gFOBT detection of simulated bleeding in the duodenum

One milliliter of blood delivered to the duodenum was detected by gFOBT with 29% sensitivity, 5 mL was detected with 86% sensitivity, and 10 mL was detected with 100% sensitivity (Table 1). Sensitivity of the gFOBT to detect blood varied significantly with delivered blood volume (Fisher‐Freeman‐Halton exact test, *P* = .02), with increased detection of larger blood volumes.

#### 3.2.3 Effect of blood volume on gFOBT detection of simulated bleeding in the colon

Sensitivity of gFOBT to detect blood delivered to the colon was 86% for 0.1 mL, 57% for 0.5 mL, and 57% for 1 mL (Table 1). There was no significant difference in sensitivity of the gFOBT to detect different volumes of blood delivered to the colon (Fisher‐Freeman‐Halton exact test, *P* = .45).

#### 3.2.4 Effect of GI site on gFOBT detection of simulated bleeding

There was no difference in gFOBT detection of any tested volume of blood when comparing the gastric and duodenal routes of administration (10 mL, 5 mL, 1 mL: Fisher‐Freeman‐Halton exact test, *P* = 1.00). There was no difference in detection of 1 mL of blood delivered to the stomach, duodenum, or colon (Fisher‐Freeman‐Halton exact test, *P* = .62). gFOBT was 86% sensitive to detect 0.1 mL of blood delivered to the colon, compared with 29% sensitivity in detecting a 10‐fold higher volume (1.0 mL) delivered to either the stomach or the duodenum (Table 1); with gastric and duodenal routes of administration collapsed into one group for analysis (upper GI), gFOBT was significantly more sensitive to detect 0.1 mL of blood delivered to the lower GI tract than to detect 1 mL of blood delivered to the upper GI tract (Fisher's exact test, *P* = .02).

### 3.3 gFOBT detection of biopsy‐associated GI bleeding

50% of animals with biopsy‐induced bleeding tested positive via gFOBT. There were three fecal samples collected for gFOBT that contained grossly visible blood; each of these instances occurred following colonic biopsy, and in each case, the sample tested positive by gFOBT.

#### 3.3.1 Effect of GI site on gFOBT detection of biopsy‐associated bleeding

Gastric biopsies were detected with 25% sensitivity, duodenal biopsies were detected with 38% sensitivity, and colonic biopsies were detected with 88% sensitivity (Table 1). With gastric and duodenal routes of administration collapsed into one group for analysis, gFOBT was more sensitive to detect lower GI tract bleeding as compared to upper GI tract bleeding (Fisher's exact test, *P* = .03).

### 3.4 Evaluation of repeated testing protocols

#### 3.4.1 Effect of repeated testing on gFOBT Sensitivity

**Figure 3:**
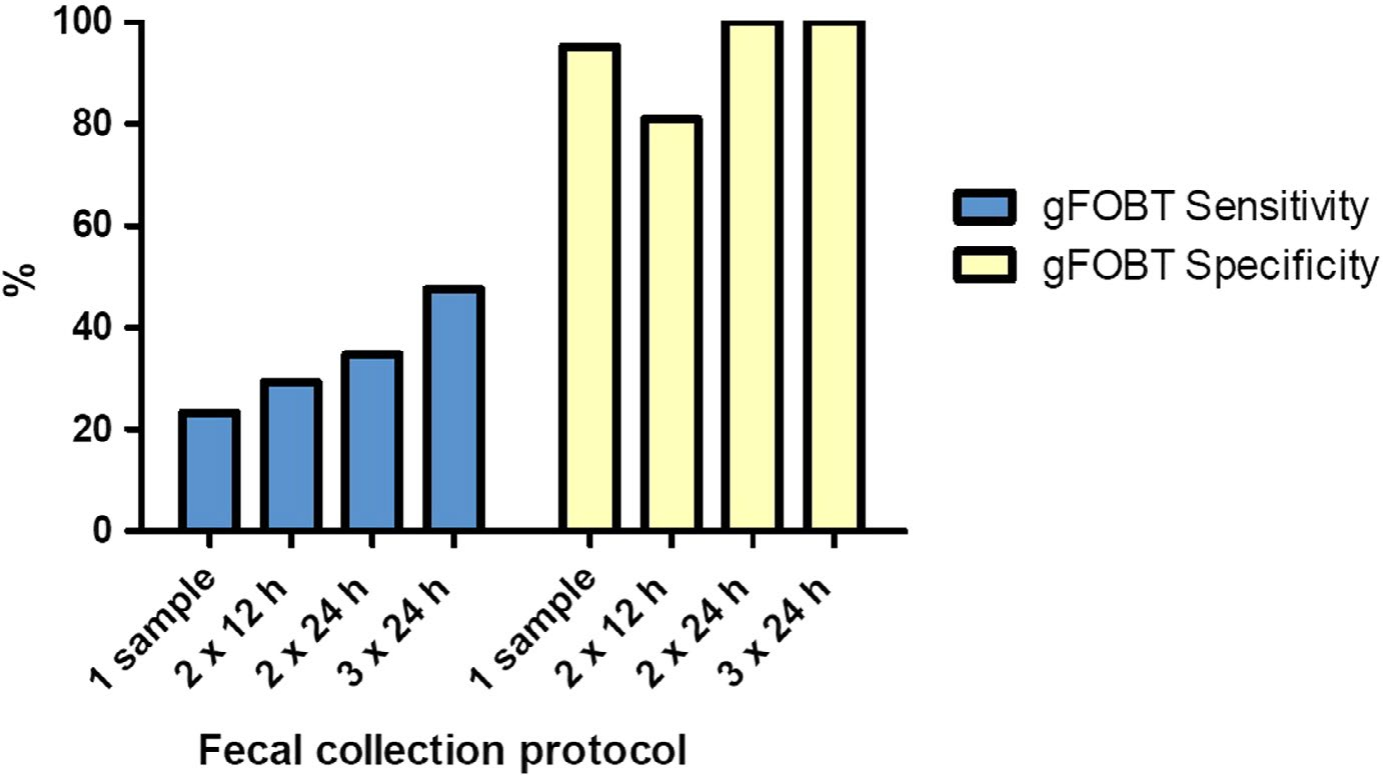
Guaiac fecal occult blood test (gFOBT) sensitivity and specificity in detection of gastrointestinal bleeding following pinch biopsies of the stomach, duodenum, and colon. Test sensitivity and specificity were evaluated on the basis of several fecal collection protocols: any single fecal collection (1 sample), any two samples collected 12 h apart (2 × 12), any two samples collected 24 h apart (2 × 24), and any three samples collected 24 h apart (3 × 24)

An additional objective of this study was to assess whether repeated gFOBT tests over time would alter sensitivity of the gFOBT to detect GI bleeding in the rhesus macaque. With data pooled following gastric, duodenal, and colonic biopsies, sensitivity to detect bleeding was 23.3% for any single test (1 sample), 29.3% for any two samples collected 12 hours apart (2 × 12), 34.7% for any two samples collected 24 hours apart (2 × 24), and 47.6% for any three samples collected 24 hours apart (3 × 24; Figure 3).

#### 3.4.2 Effect of repeated testing on gFOBT Specificity

Evaluating the negative control condition, specificity of the gFOBT in detecting bleeding was 95.2% for any single test (1 sample), 81.0% for any two samples collected 12 hours apart (2 × 12), 100% for any two samples collected 24 hours apart (2 × 24), and 100% for any three samples collected 24 hours apart (3 × 24; Figure 3).

#### 3.4.3 Evaluation of gFOBT sensitivity over time

Considering all experimental procedures, percent positive gFOBT were as follows: 46% at 24 hours, 45% at 36 hours, 35% at 48 hours, 14% at 60 hours, and 16% at 72 hours There was an effect of post‐procedural time on gFOBT sensitivity to detect occult blood over the 72 hours post‐procedural period (Kruskal‐Wallis test, *P* < .01), with significantly higher detection at 24 hours and 36 hours than 60 hours and 72 hours (Dunn's multiple comparisons tests; *P* < .05).

## 4 DISCUSSION

This study demonstrates that gFOBT can detect bleeding in both the upper and lower GI tract of macaques, with highest sensitivity to detect colonic bleeding. This finding is consistent with data reported in the human literature and supports the use of gFOBT as a screening test for GI bleeding in rhesus macaques. In our study, all tested blood volumes were detected with greater than 50% sensitivity in the colon, and the gFOBT was better able to detect blood volumes delivered to the colon that were 10‐fold lower than volumes delivered to the upper GI tract. In addition, biopsy‐associated bleeding, which was intended to more closely mimic bleeding associated with clinical disease in macaques, was detected with 88% sensitivity in the colon. In macaques, previous case reports of gFOBT use have suggested increased gFOBT sensitivity in lower vs upper GI tract bleeding. Intermittently positive FOBT were found in most cases of lower GI adenocarcinoma in one retrospective study of 32 macaques.^3^ Additionally, a single FOBT was positive in a case study of a macaque with adenocarcinoma of the ileocolic junction, mucous membrane and cutaneous pallor, and clinical anemia.^4^ No evidence of active hemorrhage or ulceration was noted on gross necropsy or histopathology <3 weeks later; this suggests that even in the absence of grossly visible hemorrhage or ulcerative lesions, blood loss from the lower GI tract can proceed at a rate adequate to outpace regenerative capabilities and to produce a positive FOBT. In a case report of gastric adenocarcinoma with accompanying lymphoplasmacytic gastritis and microcytic hypochromic anemia, a single FOBT was negative.^34^ Our study findings provide the first supportive evidence of differences in sensitivity of gFOBT based on location of bleeding in the rhesus macaque. Similarly, in humans, oral consumption of 15 mL of autologous blood per day for three consecutive days was required to produce at least one positive gFOBT result, out of three tests, in 60% of healthy humans,^23^ whereas 2‐4 mL daily blood loss from the descending colon or rectosigmoid junction was detected in 86% of samples in another study.^24^ The finding that gFOBT is more sensitive to detect lower than upper GI tract bleeding in macaques, as previously reported in humans, is reflective of the mechanism of gFOBT blood detection, which relies on the presence of the intact heme moiety. In humans, heme degradation is localized primarily to the large intestine.^35^ Therefore, we would expect that blood from the upper GI tract would be degraded prior to defecation, whereas colonic blood would to a lesser degree or not at all. Further supporting this, we found no significant differences in detection of gastric vs duodenal bleeding, either simulated or biopsy‐induced. Because the digestive environment of the stomach differs considerably from that of the duodenum,^36^ bleeding from both upper GI tract segments was evaluated in this study in order to determine whether exposure to the stomach might have a significant effect.

One limitation of this study in comparing biopsy‐associated upper and lower GI tract bleeding is that we were unable to directly visualize bleeding following blind colonic biopsies. However, histopathology of all biopsy samples was performed in order to confirm consistent depth across all groups. Histopathology findings revealed that biopsies from all procedures reached, at minimum, the depth of the muscularis mucosae, suggesting that colonic biopsy produced bleeding comparable to upper GI biopsy.

Interestingly, despite the significant increase in gFOBT sensitivity to detect 0.1 mL blood in the lower GI as compared to 1 mL blood delivered to the upper GI in the present study, we did not observe a significant difference in detection when comparing 1 mL of blood delivered to the upper vs lower GI. This result may be attributable to inherent sampling error; it is possible that a single distal colonic or rectal small volume bleed may go undetected by gFOBT due to the sporadic selection of sampling site within any given fecal bolus and the presence of blood in a presumably small region of stool. Both the simulated hemorrhage and biopsy experiments, as well as spontaneous bleeding in the clinical setting, are susceptible to this limitation. Additionally, in this study, it is possible that there was a laxative effect of the blood and saline enema (2 mL maximum total volume), eliciting prompt defecation following the procedure. Since cage cleaning was scheduled following recovery of the macaques from chemical restraint, with the first fecal collection occurring after that time, the blood‐containing fecal bolus or boluses may have been missed following colonic administration due to this aspect of experimental design. However, though our first fecal collection occurred at the 24‐hour time point, initial observations indicated that macaques did not defecate within the first 12 hours post‐anesthesia.

A second major finding of this study is that gFOBT is more sensitive to detect larger as compared to smaller volumes of blood delivered to the upper GI tract of rhesus macaques, which is again consistent with data in human subjects. In one study of healthy humans, in which feces were tested daily until 72 hours following cessation of blood ingestion, 20 mL of daily blood ingestion (60 mL total over 3 days) was required for one commercial gFOBT (Hemoccult II SENSA) to achieve a sensitivity of 64%.^25^ This blood volume corresponds to approximately 0.44% total blood volume daily or 1.3% over 3 days, calculated according to an average human total blood volume of approximately 4.5 L.^37^ In another study of healthy humans, 5 mL of blood ingestion per day for 3 days (estimated 0.11% total blood volume daily or 0.33% in 3 days) was not detected in any participants.^23^ In our study, gFOBT sensitivity was similarly volume‐dependent in the upper GI tract; in the stomach and duodenum, the gFOBT more readily detected larger as compared to smaller volumes of simulated bleeding. Ten mL of blood, approximately 1.2%‐1.5% total blood volume in our study subjects,^38^ was detected with 100% sensitivity, while 1 mL of blood (approximately 0.12%‐0.15% total blood volume) was detected with just 29% sensitivity. Although it is known that various GI diseases produce bleeding, there are no known published data describing quantity or frequency of bleeding associated with different conditions. One study of humans with known hemorrhagic upper GI tract lesions found 26% sensitivity of gFOBT to detect blood.^23^ Given that 1 mL of blood administered to the upper GI tract of macaques in the present study was detected with a similar 29% sensitivity, approximately 1 mL of blood may be reflective of an average hemorrhage volume in the rhesus macaque. Furthermore, gFOBT detection of biopsy‐associated bleeding was similarly 25% (stomach) and 38% (duodenal) in the upper GI tract in this study, suggesting that blood loss from partial‐thickness tissue biopsies, mimicking pathologic ulceration, was in the approximate range of 1 mL.

Unlike in the upper GI tract, however, rate of blood detection did not vary significantly with volume in the lower GI tract of macaques in our study. One possible reason for this lack of observed effect is that we did not reach the lower limit of detection with the tested blood volumes. gFOBT detected our smallest lower GI‐delivered blood volume, 0.1 mL, in 86% of macaques, similar to the 88% detection of biopsy‐induced colonic bleeding. It is possible that we may have been able to identify a lower limit of detection if even smaller blood volumes had been tested in the colon. Another possible but unlikely factor preventing blood volume‐dependent detection in the lower GI tract, as discussed above, could be the presence of a laxative effect in the colon following administration of blood, leading to loss of introduced blood boluses prior to fecal sample collection. If present, this effect would be expected to be more pronounced at the greater delivered blood volumes and would thus provide an explanation for the apparent decreased ability of gFOBT to detect 0.5 and 1 mL of blood in comparison with the 0.1 mL blood volume.

Our study demonstrated a gFOBT specificity of 95.2% when used to assess the presence of blood in a single fecal sample, and 81%‐100% when performed using repeated sampling protocols in adult male rhesus macaques. Similarly, specificity of gFOBT in humans is reported at 77.9%‐98.8% in screening populations.^39^, ^40^ False‐positive results can be caused by high dietary peroxidases (eg, broccoli, cauliflower, horseradish, and melons)^41^ or animal‐derived heme (ie, red meat)^42^; additionally, NSAIDs and anticoagulant medications have been shown to increase the rate of false‐positive results.^43^, ^44^ False‐negative results can be caused by ingestion of product high in vitamin C (eg, citrus fruit).^45^ However, a recent systematic review of gFOBT use in humans concluded that available data do not support dietary restrictions when screening for colorectal cancer.^46^ The presence of interfering compounds, when consumed in a typical amount, is generally not great enough to significantly alter sensitivity or specificity of the test; furthermore, compliance with gFOBT testing in humans is decreased when dietary restrictions are imposed.^46^ Regardless, for gFOBT use in animals, it is easy to ensure compliance with the dietary restrictions necessary for maximum sensitivity and specificity of gFOBT, and compliance with completing gFOBT testing is not a factor. At our institution, a commercial diet containing 910 mg/kg ascorbic acid is supplemented in a controlled manner with a variety of food enrichment items. We do not provide any meat products, and it is easy to avoid potentially confounding produce (ie, items containing high peroxidase or high ascorbic acid). Therefore, for typical use of the test, most factors that can lead to false‐positive or false‐negative results can be eliminated in captive animals.

In the present study, macaques were excluded from undergoing procedures following a positive pre‐procedural gFOBT test. Positive pre‐procedural tests occurred following procedures in both experiments (Experiment 1:7.9%; Experiment 2:8.3%) and at a frequency comparable to the single‐sample false‐positive rate of 4.8%. These results suggest that positive gFOBT results in these cases were false positives, rather than indicating detection of continued bleeding.

Evaluation of different sampling regimens in our biopsy model revealed that repeated use of gFOBT in rhesus macaques produced increased sensitivity to detect bleeding as compared to a single time point test. Evaluation of repeated testing regimens was limited to the biopsy model study groups as this degree of GI bleeding is thought to most closely recapitulate clinical bleeding associated with most common GI diseases in macaques. Overall gFOBT sensitivity progressively increased with multiple fecal collections, with highest sensitivity to detect biopsy‐associated GI bleeding using three fecal samples collected 24 hours apart. Together with a specificity of 100% using this protocol in the present study, we recommend that gFOBT be performed using three sample time points collected 24 hours apart in cases of suspected GI bleeding or for screening of susceptible populations. For colorectal cancer screening using gFOBT in humans, sampling of two fecal sections from three consecutive fecal samples (six total samples) is widely recommended.^47^ This recommendation was formed according to the notion that lesions may bleed intermittently or that blood may be ill‐distributed throughout the stool.^48^ Though the recommendation for human use relies upon consecutive fecal samples rather than on timed collections, our current recommendation for gFOBT use in rhesus macaques is largely in agreement; furthermore, timed collections are more practical in the veterinary clinical setting, where collection of consecutive samples is often not feasible.

Guaiac fecal occult blood testing is a widely used diagnostic tool for detection of GI tract bleeding in rhesus macaques, owing to its non‐invasive nature and ease of use in combination with the high prevalence of GI tract disease in these species. However, the test has historically been used in rhesus macaques and other non‐human primates without evidence‐based usage or interpretation guidelines. This study is the first to demonstrate that gFOBT is well suited for use as a screening test in rhesus macaques and to establish a recommendation for a repeated testing regimen. Further investigation of gFOBT performance in macaques affected by spontaneous disease would be invaluable in further defining the utility of gFOBT in the clinical setting.

## CONFLICT OF INTEREST

The authors declare no conflicts of interest.
